# An unusual case of chronic abdominal pain: an association between Celiac disease and Crohn’s disease

**DOI:** 10.1093/omcr/omab008

**Published:** 2021-04-28

**Authors:** Leen Jamel Doya, Maria Naamah, Noura Karkamaz, Narmin Hajo, Fareeda Wasfy Bijow, Ali Ibrahim, Rana Issa

**Affiliations:** 1 Department of Pediatrics, Tishreen university hospital, Lattakia, Syria; 2 Department of Pediatrics, Professor of Gastroenterology and Hepatology, Tishreen university hospital, Lattakia, Syria; 3 Department of Pathology, Professor of pathology, Tishreen university hospital, Lattakia, Syria

## Abstract

Inflammatory bowel diseases (IBD) and Celiac disease (CeD) are immune-mediated gastrointestinal diseases with incompletely understood etiology. Both diseases show a multifactorial origin with a complex interplay between genetic, environmental factors, and some components of the commensal microbiota. The coexistence of celiac disease with Crohn’s disease is rarely reported in the literature. Here, we report a case of a 13-year-old Syrian male who presented with a history of abdominal pain, anorexia and pallor. CeD and Crohn’s disease was documented on gastrointestinal endoscopy and histological study. The patient was treated with a gluten-free, low fiber, high caloric diet, and a course of oral corticosteroids with an improvement in growth rate and abdominal pain.

## INTRODUCTION

Celiac disease (CeD) is a genetically determined chronic inflammatory disease of the small intestine. It occurs in about 1% of the population worldwide [1]. The pathogenesis of CeD is multifactorial but is mainly defined by the presence of 3 factors: genetic changes, exposure to gluten and an altered immune response [2]. It is associated with other autoimmune diseases such as autoimmune thyroiditis, type I diabetes, Addison’s disease, primary biliary cirrhosis, and inflammatory bowel disease (IBD) [3]. The prevalence of IBD in CeD is estimated to be (1–3.2)% that is (3–10) times higher than the prevalence of IBD in the general population [4]. Compared to adults, the coexistence of CeD with CD in children is extremely rare. To our knowledge, no more than 30 cases of CeD with CD in children have been reported in the literature, most of them were more than 12 years old.

## CASE REPORT

**Figure 1:**
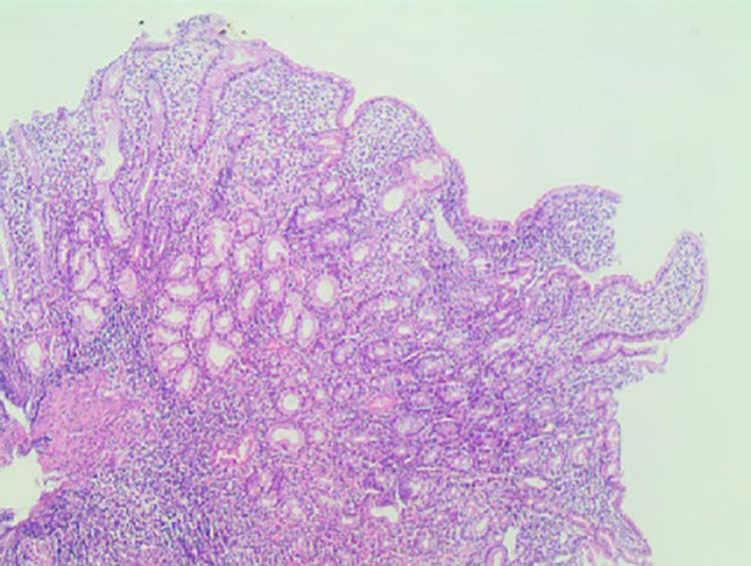
Compatible with grade (3a) on the Marsh grading system for CD classification.

A 13-year-old Syrian male presented to the pediatric gastroenterology clinic with a history of recurrent episodes of epigastric and peri-umbilical abdominal pain for 3 years, which were exacerbated by food and partly controlled with analgesics. The episodes were associated with anorexia and pallor. There was no significant past medical or family history. During that period, the child was diagnosed with iron deficiency anemia [hemoglobin (Hb) 8.9 g/dl, Mean corpuscular volume (MCV) 69 fl] with no response to iron supplemental therapy. Anti-gliadin antibodies IgA, IgG (AGA IgA, IgG), Anti-tissue transglutaminase Antibodies IgA (anti-TTG IgA) were markedly elevated (more than 100 U/mL). An Esophagogastroduodenoscopy (EGD) had been performed a year pirior to admission, with normal findings, and the duodenal biopsies had shown CeD type 3a according to the Marsh classification (partially atrophic villi with lymphocytic exocytosis at 56 lymphocytes/100 enterocytes in the lamina properia) (Fig. 1). The patient was treated with a gluten-free diet without improvement in abdominal pain.

**Figure 2:**
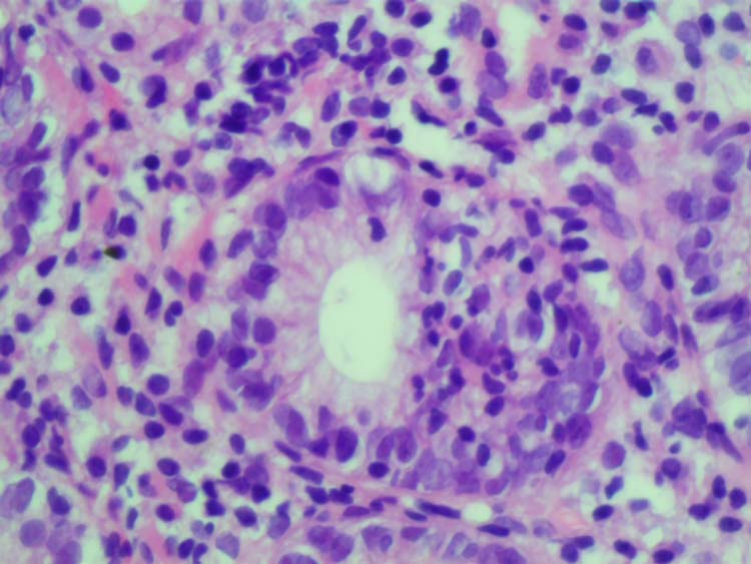
Microscopic image: stomach abcesses.

**Figure 3:**
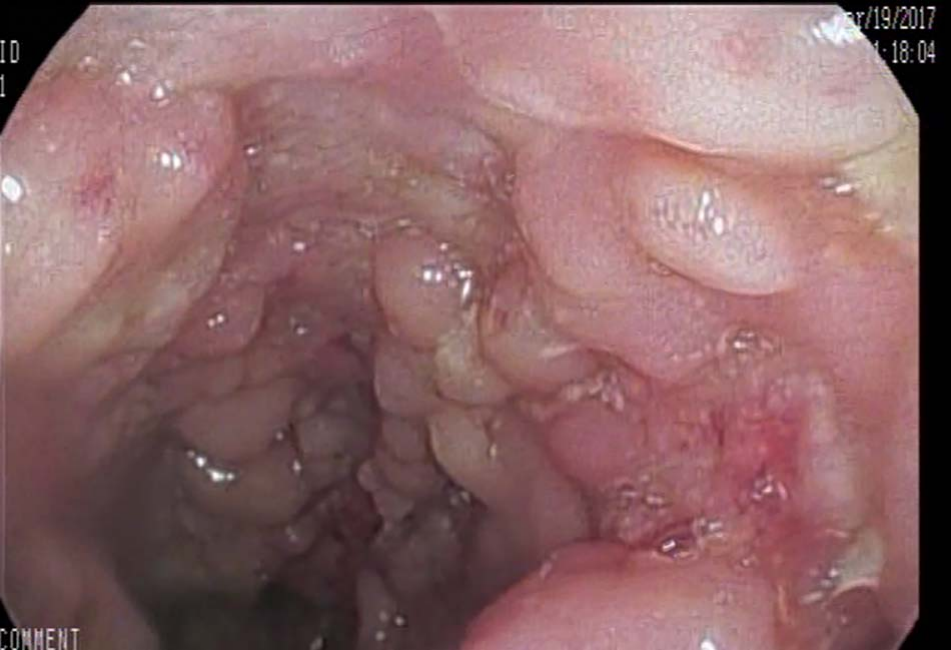
Longitudinal ulcers alternating with healthy mucosa.

**Figure 4:**
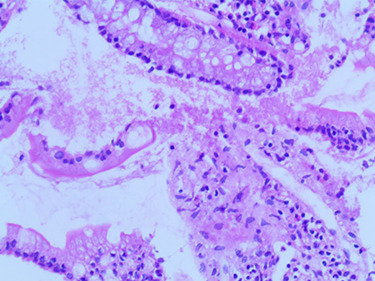
Histological ileal biopsies: small histiocytes aggregates.

On physical examination, his body weight was 23 kg (SD –4) and his height was 137 cm (SD –2). He was pale, but his observations were stable and he appeared generally well. The patient underwent multiple investigations that showed hypochromic microcytic anemia [Hb 8.6 g/dl, MCV 68 fl] associated with an inflammatory respond [C- reactive protein (CRP) 81 mg/l, erythrocyte sedimentation rate (ESR) 40 mm at the end of 1^st^ hour] and hypoalbuminemia (Alb 3,2 g/dl). AGA (IgA, IgG) and anti-TTG IgA were within the normal range on this admission. Investigations including hepatic and renal function, blood glucose, urinary investigations and an abdominal ultrasound were all normal. Therefore, an EGD was repeated showing diffuse scattered nonbleeding ulcers, covered with fibrinous membranes in the esophagus, antrum, and duodenum. Histological examination of stomach biopsies showed chronic active ulcerated gastritis with diffuse lymphocytic infiltration in the lamina properia and stomach abscesses (Fig. 2). Duodenal biopsies showed uncertain chronic duodenal mucosa inflammation. The ileocolonoscopy showed regions of congestive mucosa in the rectum, colon, caecum, and ileum with longitudinal ulcers alternating with healthy mucosa (Fig. 3). Histological study of colonic and ileal biopsies showed focal erosions, crypitis, crypt abscesses with fissures, and non-caseating granulomas which correlates with active Crohn’s disease (Fig. 4).

The patient was treated with a gluten-free, low fiber, high caloric diet, and a course of oral corticosteroid (Prednisolone 2 mg/kg/day) with progressive tapering after 3 weeks.

After about one month of initiation treatment, the patient had significant clinical improvement. His symptoms improved markedly, and he gained 3 kg in weight. We then added 5-aminosalicylic acid (Mesalamine) 25 mg/kg/day, vitamins, and iron supplements.

We followed the patient for 3 years after initial treatment. During these years, the steroid was gradually reduced until it was discontinued after 6 months of treatment at full dose. Currently, the patient is treated with a gluten-free, low fiber, high caloric diet, mesalamine 25 mg/kg/day and vitamins. His CD remains in remission and he now has a normal growth rate with normal pubertal development.

## DISCUSSION

The first report on the occurrence of CeD and CD in the same patient was in 1967 [5]. The underlying coexisting mechanism of the two diseases is still controversial. Both are genetically predisposed diseases that share a similar immune-mediated Th1 inflammatory cascade response to the loss of tolerance to external antigens (gluten for CeD and intestinal flora to CD) [6]. Symptoms of malabsorption, diarrhoea, abdominal pain, weight loss, and long-standing history of iron deficiency anaemia are common in both CD and CeD, which can present a challenge in distinguishing between the two conditions. Biochemical changes would also be similar and therefore not very helpful in making the diagnosis [7]. Malabsorption and anaemia in celiac patients that have no response to treatment are investigated for possible IBD as the 2 conditions may coexist [8]. Several patients with CD and CeD are seronegative for AGA, anti-endomysium antibodies, and anti-TTG IgA particularly patients showing slight/moderate histological damage of duodenum (Marsh II-IIIa lesions) [9]. In our case, the serological tests for CeD were positive, although the patient had Marsh IIIa lesions.

Although interestingly, there is some overlap. A toothcomb appearance in the duodenum is one of the most characteristic endoscopic and histological findings in CD, and it can also be present in CeD, with villous atrophy seen in up to 20% of patients with CeD [10].

In our case, the dominant symptom in our patient was chronic abdominal pain for the last three years, resulting in malnutrition, serious weight loss, and anaemia without any response to appropriate therapy. Colonoscopy examination was performed, which revealed the coexistence of CeD and CD. The patient was treated with improvement in abdominal pain, weight, and anaemia.

## CONCLUSION

Co-existent disease should be kept in mind when the diagnosis is not clear or the patient is not improving on appropriate therapy. Paediatricians must consider the investigation of CD in patients with CeD, who have poor control of symptoms despite receiving adequate diet.

## Data Availability

All data generated or analyzed during this study are included in this published article.
